# An Ideal Business and Social Meeting of Dentists

**Published:** 1895-02

**Authors:** 


### Editorial, Etc.

AN ideal business and social meeting of dentists was held in Washington, January 12, 1895. Last Otober members of the Maryland State Dental Association and of the Washington City Dental Society met in Baltimore, gave clinics and discussed them. About thirty dentists from Washington attended and were entertained at the Eutaw House. As the first clinic was a success, a second meeting for clinics was decided upon. It was held in the Dental Department of the Columbian University, Washington, D. C., and was under the direction of Dr. Williams Donnally of that city. Those who gave clinics from Maryland were Drs. Cyrus M. Gingrich, Wm. A. Mills, E. E. Cruzen, David Genese, Wm. Shaw Twilley, L. B. Wilson, C. C. Harris; and from Washington Drs. W. St. George Elliott, W. E. Dieffenderfer, J. L. Wolf, E. A. Bryant, H. B. Noble.

In the evening a complimentary banquet was given by the Washington City Dental Society, at which no papers were read, but the demonstrations of the day were discussed and responsive remarks of a lighter vein were made by dentists, and by a layman present who talked from the standpoint of a patient. The dinner was in charge of a committee composed of Drs. H. M. Schooley, chairman; Wm. Donnally, and J. Roland Walton, the latter presiding and calling upon the speakers. More than seventy dentists attended, the number about equally divided between Maryland and Washington City. It is understood that arrangements have been made for a still larger gathering in Baltimore within this year. R. G.

